# Evaluation of Cocaine Effect on Endogenous Metabolites of HepG2 Cells Using Targeted Metabolomics

**DOI:** 10.3390/molecules26154610

**Published:** 2021-07-29

**Authors:** Adamantios Krokos, Olga Deda, Christina Virgiliou, Helen Gika, Nikolaos Raikos, Eleni Aggelidou, Aristeidis Kritis, Georgios Theodoridis

**Affiliations:** 1 Department of Chemistry, Aristotle University of Thessaloniki, 54124 Thessaloniki, Greece; akrokosa@chem.auth.gr (A.K.); cr_virgi@hotmail.com (C.V.); 2 Laboratory of Forensic Medicine and Toxicology, Department of Medicine, Aristotle University of Thessaloniki, 54124 Thessaloniki, Greece; oliadmy@gmail.com (O.D.); gkikae@auth.gr (H.G.); raikos@auth.gr (N.R.); 3 Biomic_AUTh, Center for Interdisciplinary Research and Innovation (CIRI-AUTH), Balkan Center, B1.4, 10th km Thessaloniki-Thermi Rd, P.O. Box 8318, 57001 Thessaloniki, Greece; 4 Laboratory of Physiology and Pharmacology, School of Medicine, Aristotle University of Thessaloniki, 54124 Thessaloniki, Greece; angelide@auth.gr (E.A.); kritis@auth.gr (A.K.)

**Keywords:** cocaine toxicity, targeted metabolomics, HepG2, liquid chromatography, mass spectrometry

## Abstract

Cocaine toxicity has been a subject of study because cocaine is one of the most common and potent drugs of abuse. In the current study the effect of cocaine on human liver cancer cell line (HepG2) was assessed. Cocaine toxicity (IC50) on HepG2 cells was experimentally calculated using an XTT assay at 2.428 mM. The metabolic profile of HepG2 cells was further evaluated to investigate the cytotoxic activity of cocaine at 2 mM at three different time points. Cell medium and intracellular material samples were analyzed with a validated HILIC-MS/MS method for targeted metabolomics on an ACQUITY Amide column in gradient mode with detection on a triple quadrupole mass spectrometer in multiple reaction monitoring. About 106 hydrophilic metabolites from different metabolic pathways were monitored. Multivariate analysis clearly separated the studied groups (cocaine-treated and control samples) and revealed potential biomarkers in the extracellular and intracellular samples. A predominant effect of cocaine administration on alanine, aspartate, and glutamate metabolic pathway was observed. Moreover, taurine and hypotaurine metabolism were found to be affected in cocaine-treated cells. Targeted metabolomics managed to reveal metabolic changes upon cocaine administration, however deciphering the exact cocaine cytotoxic mechanism is still challenging.

## 1. Introduction

Methylbenzoylecognine, commonly known as cocaine is a major alkaloid of *Erythroxylon coca* Lam. Its structure is presented in Supplementary Materials Figure S1. Cocaine’s metabolic fates in humans include primarily a rapid hydrolytic metabolism to benzoylecgonine (BE) and ecgonine methyl ester (EME) and secondary to norcocaine, which in turn is metabolized to N-hydroxynorcocaine, norcocaine nitroxide, and finally, to the hepatotoxic norcocaine nitrosonium ion [1,2]. The production of reactive oxygen species (ROS), the depressed mitochondrial respiration, and the diminishing antioxidant system due to the depletion of intracellular and mitochondrial glutathione have been linked to cocaine-induced in vivo and in vitro hepatotoxicity [3]. A dose, sex, and enzymes activity-dependent cocaine metabolism explains the variations of the hepatic tissue damage and the differential hepatic zonal necrosis [4]. The exact mechanism by which cocaine metabolites cause toxicity on target tissues and how this is linked to fatal conditions, are still under investigation [5]. For both human and animal models in multi-organs and systems such as heart, liver, kidney, and central nervous system (CNS), cocaine-induced toxic effects are linked to oxidative stress [6].

Cocaine is an illegal highly addictive drug with a high estimated prevalence of use. Its consumption leads to euphoria and hyper-alert state of mind, by inhibiting catecholamine re-uptake and flooding the synaptic cleft [7,8]. The mechanism of addiction to dependence-producing drugs certainly remains a global issue due to the complications related to its abuse, varying from myocardial ischemia and infarction, brain, liver, and kidney damage, and death [1]. Drug-dependence describes a complex situation in which many factors are involved, composing the mechanism of dependence formation [9]. Harmful effects of cocaine are further promoted by the use of street drug contaminants resulting in enhanced toxicity [8].

Modern analytical technologies empower the reliable detection of major addictive drugs and their metabolites in various biospecimens facilitating forensic purposes [10,11,12,13,14,15,16,17]. Metabolomics, an “-omics” approach, was lately applied also to drug addiction studies, [18] offering advanced tools to investigate the biochemistry of drug toxicity mechanisms, drugs dependence, treatment, withdrawal, or relapse [9,19]. Metabolic profiling aims to capture the holistic effect that a stimulus induces on the cellular metabolism through the detection of endogenous metabolites [20,21,22]. Employing modern analytical platforms, the influence of several determined factors to drug toxicity and abuse such as dose and route of administration [23] can be quantified through the measurable cellular response.

Despite the breadth of published studies on cocaine’s in vivo and in vitro effect, a few of them engage metabolomics [9,24,25,26,27]. In the present study the cytotoxic in vitro effect of cocaine administration was investigated through metabolomics-based analyses of both intracellular and extracellular material of HepG2 cell line, for the first time. A validated targeted LC-MS/MS method [28] was used to detect and quantify metabolic changes in response to cocaine administration from a panel of low molecular weight primary metabolites. The decoding of the mechanism of cocaine hepatotoxic effect through metabolomics may lead to new therapeutic approaches.

## 2. Results

### 2.1. XTT Cell Proliferation Assay

**Figure 1:**
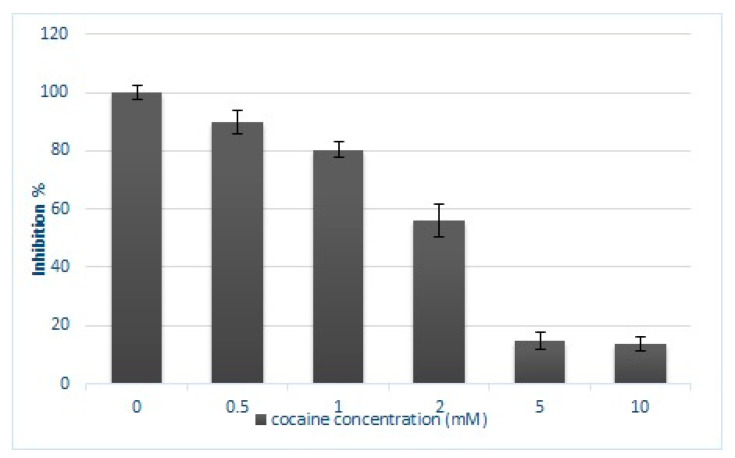
Cell inhibition of HepG2 cells exposed for 24 h to different concentrations (0.5, 1, 2, 5, and 10 mM) of cocaine. Data are expressed as a percentage of the control value and the values are presented as means ± SD of three independent experiments, each made in triplicate.

Cocaine cytotoxicity has been studied using an XTT assay to experimentally evaluate the half-maximal inhibitory concentration (IC50), (see Section 4.1 XTT assay). Hepatocytes were exposed to increasing concentration of cocaine (0.5 mM, 1 mM, 2 mM, 5 mM, and 10 mM) for 24 h and the percentage of inhibition was determined (Figure 1). A dose-dependent inhibition decrease was observed in comparison to the control. The IC_50_, was estimated to be 2.428 mM.

### 2.2. Cell Growth Rate

**Figure 2:**
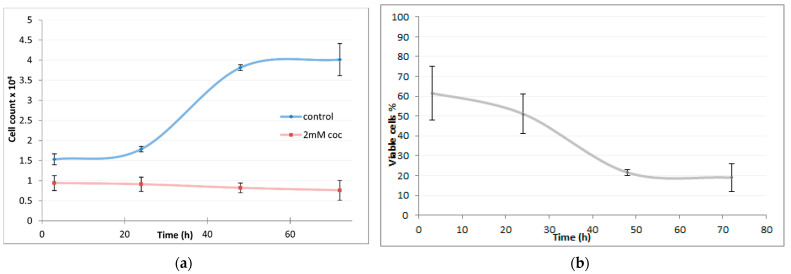
(**a**) Number of cells count vs. time upon treatment with 2 mM of cocaine; blue line corresponds to control cell culture; red line corresponds to treated culture with 2 mM of cocaine. (**b**) Percentage of viable cells treated with 2 mM of cocaine over time. The values are presented as means ± SD shown with error bars.

After cocaine IC_50_ was calculated, cells growth rate was determined after up to 72 h of HepG2 cultivation with 2 mM cocaine. In Figure 2 (left), a clear growth inhibition can be seen after 24 h incubation in the treated cells. Cell viability was found to decrease up to twenty per cent after 24 h treatment with 2 mM cocaine as can be observed in Figure 2 (right). After 48 h or 72 h a significant 80% reduction of cell viability was observed.

### 2.3. Microscopic View of HepG2

**Figure 3:**
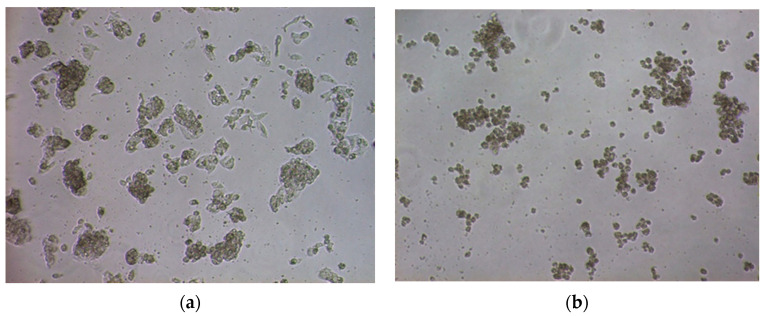
(**a**) Microscopy image of control HepG2 cells; (**b**) image of 10 mM cocaine-treated HepG2 cells (magnification 10×).

Cell’s morphology monitored by a microscope provided indication of cocaine apoptotic effect [29]. The untreated cells exhibit an epithelial-like morphology. They were attached to the bottom of the well, with clear and abundant cytoplasm, and were growing in clusters. The cells treated with 10 mM cocaine appeared smaller, condensed, and they had void in the cytoplasm and detached easily from the well. In Figure 3 microscopy images of HepG2 control cells (a), and HepG2 cells treated with 10 mM of cocaine (b) are provided. 

### 2.4. Targeted Metabolic Profiling Analysis

A targeted metabolic profiling approach was performed to investigate the metabolic changes induced in HepG2 cells after cocaine treatment. A validated targeted HILIC-MS/MS method was applied in the analysis of intracellular and cell medium samples. The method performs the determination of 106 endogenous metabolites such as amino acids, purines, carbohydrates, vitamins, amino acids derivatives, and other small molecules.

With the profiling method developed to be applied for the determination of key metabolites [28], metabolic perturbations induced by cocaine could be investigated. From the circa 100 metabolites that the method monitors, 41 metabolites were detected and quantified in the intracellular material, whereas 55 metabolites were detected and quantified in the cell culture media. 

Before data analysis, inspection of Quality Control (QC) samples was performed as a typical procedure for the assessment of the analytical precision to ensure that any variations in the metabolic content of treated vs. control samples were due to intervention. Only compounds with RSD less than 30% were further investigated. 

In Table 1, the mean concentration found in the intracellular material for every metabolite detected in control and treated intracellular material is given for every time point. The *p*-values and the log fold change has been calculated in every time point for the concentrations in the treated over the control cell cultures. In most of the cases, a remarkable decrease in the concentration of the metabolites can be seen in the cocaine-treated cells, whereas in very few cases a fluctuation was observed between the time points. What was concluded based on the results is that more pronounced alterations in the concentrations of the metabolites were observed after 48 h of incubation. 

**Figure 4:**
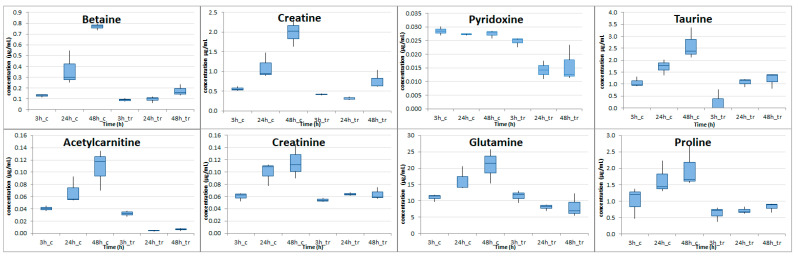
Boxplots illustrating the concentrations of metabolites with *p* < 0.05 at 24 h and 48 h in the intracellular material of cocaine-treated HepG2 cells compared to the control HepG2 cells (c stands for control, tr for cocaine-treated cells).

Overall, from the 23 amino acids and derivatives, 7 organic acid and derivatives, 5 vitamins, 3 pyrimidines, 2 sugar alcohols, and 1 purine that were determined, 1 vitamin, 4 organic acids and 9 amino acids were found with *p*-value at 48 h ranging from 0.00007 (Betaine) to 0.044 (glutamic acid). Log2FC was found to negatively increase over time in those 14 compounds and it presented the negative highest value at 48 h time point ranging from −4.12 (acetylcarnitine) to −0.80 (pyridoxine). Serine was the only compound that showed a decrease in cocaine-treated cells, compared to control cells after 48 h. Notably, at this time point serine levels were greatly decreased almost seven times (Log2FC = −2.78). All the other 13 compounds showed a decrease starting from the 24 h. Eight (8) metabolites were found with *p*-value less than 0.05 in 24 h and in 48 h and their boxplot at all time points can be seen in Figure 4.

**Figure 5:**
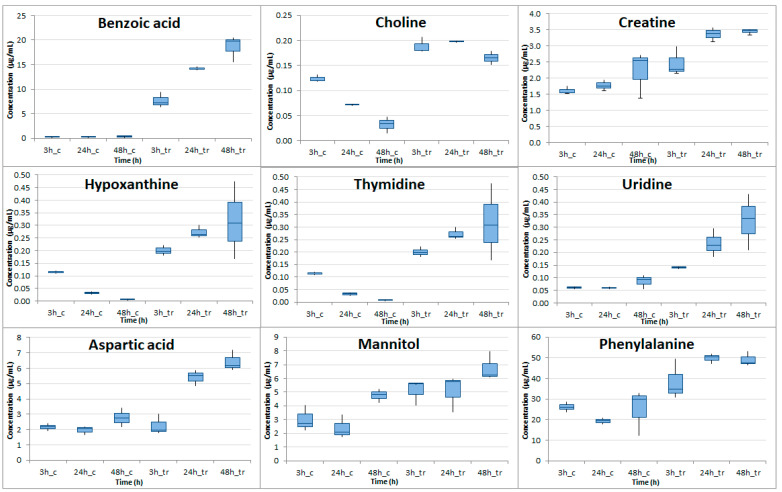
Boxplots presenting the concentrations of compounds with *p* < 0.05 at least at 24 h and 48 h in the cell medium of cocaine-treated HepG2 cells compared to control HepG2 cells (c stands for control, tr for cocaine-treated cells).

Similarly, more pronounced alterations were also detected in the cell medium of the treated samples in comparison to the control after 48 h. In Table 2, the average concentration, the *p*-value, and the Log2FC found in the cell medium for every metabolite detected in control and in cocaine-treated cell in every time point are given. Benzoic acid, choline, thymidine, uridine, hypotaurine, hypoxanthine, and creatine had *p*-value under 0.05 in all time points. In all cases an increase was observed in contrast to intracellular contents with eleven metabolites showing the lowest *p*-values at 48 h. Nine metabolites were found with *p*-value less than 0.05 in 24 h and in 48 h and their boxplot at all time points can be seen in Figure 5. Benzoic acid had the highest Log2FC ranging from 5.33 (3 h) to 6.19 (48 h). Hypoxanthine’s Log2FC greatly increased from 0.80 (3 h) to 3.07 (24 h) to 5.52 (48 h).

Overall amino acids and derivatives, pyrimidines, organic acids, and vitamin were found to be affected with cocaine treatment in both intra and extracellular material.

### 2.5. Multivariate Analysis

Using multivariate analysis through principal component analysis (PCA) no clear trend could be seen for intracellular metabolites versus incubation time in the cocaine-treated cells (samples collected in different time points: 3 h, 24 h, 48 h after cocaine treatment). 

**Figure 6:**
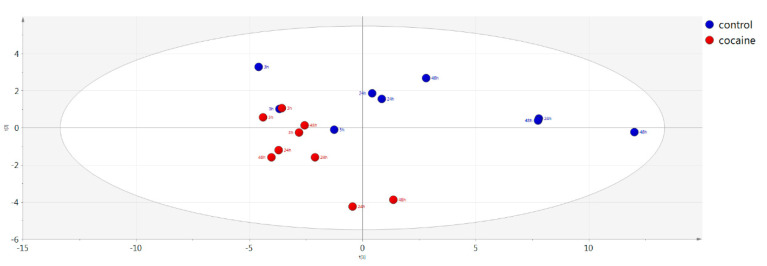
PCA model showing control (blue) vs. cocaine (red) treated intracellular material.

A clear discrimination, however, was found between control and cocaine-treated intracellular components using PCA, as shown in Figure 6, where the control samples are colored in blue and cocaine-treated in red. It can be seen that the track that is observed for the control samples with time is not followed by the treated samples which are showing a more “static metabolome”. In Figure S2 the QC samples can be seen (blue) in PCA while the other samples are colored in grey. The samples were also examined with OPLS-DA and the constructed model is shown in Figure S3 (in blue color the control and in red the cocaine-treated samples) with the pCV-ANOVA of the model found to be 0.003. The model characteristics from PCA and OPLS-DA multivariate analysis can be found in Table 3.

**Figure 7:**
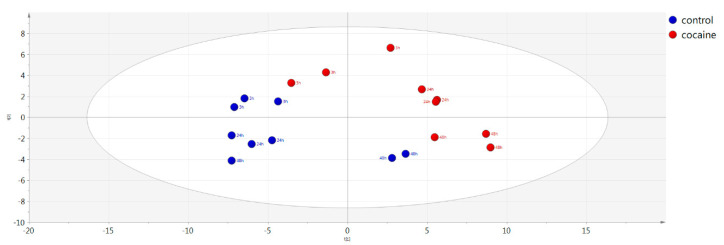
PCA model showing control (blue) vs. cocaine-treated (red) cell medium samples.

From the multivariate analysis of cell medium clear separation between the control and the cocaine-treated samples were observed. The constructed PCA model can be seen in Figure 7, where control samples are colored in blue and the cocaine-treated samples in red. The QC samples clustering in the constructed PCA model can be seen in Figure S4 in blue while in grey are depicted the control and cocaine-treated samples. The constructed OPLS-DA model of the control (blue) vs. the cocaine-treated (red) cell medium samples can be seen in Figure S5. The pCV-ANOVA of the model was calculated at 0.001. The model characteristics from PCA and OPLS-DA multivariate analysis can be found also in Table 3.

## 3. Discussion

Reasonable restrictions on the use of animals make in vitro cell cultures an optimal solution in the study of xenobiotic metabolism and toxicity [30]. Herein, a metabolomics-based investigation of cocaine’s administration effect on HepG2 cells is presented using a validated HILIC-MS/MS method. To the best of our knowledge, this is the first study that attempts to monitor cocaine impact on endogenous metabolism of HepG2 cell line using a metabolomics-based approach. The applied targeted MS method was successfully used in previous publications of our group to study the metabolic response of acute and chronic exposure to ethanol on murine urine, fecal samples, and brain tissue extracts [31,32].

The evaluation of cytotoxicity was performed using a XTT cell proliferation assay and IC50 was estimated at 2.428 mM. Hepatotoxicity of cocaine was previously evaluated at primary monolayer cultures of rat hepatocytes and hepatoma cell lines (FaO and HepG2) using LDH activity, total attached cellular protein content, and MTT reduction assay [33]. As reported the observed cocaine IC50 ranged from 1.71 mM to 2.90 mM, depending on the applied method. Higher IC50 at 6.76 mM after 24 h of cocaine exposure on C6 glioblastoma cells and lower IC50 at 4.30 mM for primary neuronal cultures were evaluated using MTT assay [8,34] and trypan blue exclusion assay [8]. In a recent study LC50 of cocaine was calculated at 2.605 mM at 48 h end point [35] suggesting that PC12 cells were more sensitive to cocaine’s activity compared to C6 astroglia-like cells from the authors’ previous study [36]. 

The response of two human hepatic tissue cultures HepG2 and WRL-68 to cocaine was evaluated using GC-MS where HepG2 cell line was proven to be more suitable than WRL-68 cell line to study in vitro cocaine metabolism and toxicity [30]. MTT assay revealed that toxicity is dose-dependent and irreversible at cocaine concentration greater than 5 mM for more than 3 days incubation. The production of the “toxic” metabolites of cocaine such as norcocaine occurred in higher doses and a diminished metabolic efficacy affect the number of viable cells. 

Based on the present study, cell viability was decreased gradually over time upon cocaine treatment in a dose near to IC50; cell population decreased by 70% approximately at 48 h. Furthermore, when HepG2 cells were treated with high cocaine concentration of 10 mM, the cells lose their membrane integrity and epithelial-like morphology.

Previous studies in neuronal cells suggest that even low cocaine concentration of 1 mM affected the membrane potential, although the mitochondrial activity remained intact [35]. Since cytotoxicity in various cell types triggered by cocaine administration targets first and foremost mitochondria, a shift to anaerobic respiration is probable [35]. 

In Table 1, significantly altered metabolites from intracellular material are illustrated separately for each time point and interesting results arose. When the 3 h cocaine’s effect on studied cells was evaluated, only 7 metabolites appeared reduced compared to the controls. The three altered metabolites, namely betaine, creatine and pyridoxine, remained differentiated at 24 and 48 h. This probably indicates that the toxic effect of cocaine acted very early on the related metabolic pathways. Changes in choline, betaine, and creatine are likely to imply a perturbed glycine, serine, and threonine metabolic pathway. 

The 24 h effect of cocaine was associated with a small increase in differentiated metabolites, while three of them continued to be differentiated at the last time point. The 48 h incubation under the influence of cocaine was accompanied by a doubling of the differentiated metabolites compared to the first two time points, probably indicating a time-dependent toxicity. These metabolites are involved in the perturbed metabolism of glycine, serine, and threonine from the first and in arginine and proline from the second time point. A disturbed alanine, aspartate, and glutamate as well as taurine and hypotaurine metabolism could be speculated from metabolites changed under 48 h cocaine treatment. 

The majority of the affected metabolites were decreased compared to controls regardless of time, indicating the toxic effect of cocaine on intracellular metabolism and the diminishing use of precursors (mainly amino acids) in cell energy production.

Regarding cell medium samples, the most intense changes were observed in 24 h, where the largest percentage of the detected metabolites was found to be altered. The 3 h effect was accompanied by 30% changes of the detected metabolites while the lowest percentage of alterations was observed at 48 h (Table 2). 

A potential meaning of the above findings could be the intense exchange and excretion of substances in 24 h, when a sharp decrease in the number of cells was observed according to the administered dose. Regarding the trend, most of the compounds increased in cell medium compared to controls as expected from the reduction observed in the intracellular material.

Benzoic acid was found to strongly augment in the treated cells while it had also a gradual increase at three time points since it is involved in the cocaine’s hepatic metabolism and can be formed by both EME and norcocaine [37]. 

By employing multivariate statistics, both unsupervised and supervised constructed models did not manage to depict the effect of the collection time points on intracellular metabolome.

Cocaine treatment was perhaps a more crucial parameter on the studied metabolome compared to the time of treatment. Although fluctuations of significantly altered metabolite quantities were observed according to the time point, in most cases these variations failed to be statistically significant across the cocaine-treated cells using univariate statistics. For this reason, further statistical analysis was conducted considering only cocaine administration on the cell line, as another approach of data interpretation. 

The employed targeted metabolomics-based method revealed a clear separation of cocaine-treated HepG2 cells from those treated only with PBS 1%, illustrated in the constructed PCA score plot. Careful examination of the PCA score plots (Figure 6) revealed cocaine-treated cells and control were clustered closely in 3 h while they moved away at the next 2 time points. Furthermore, an interesting fact was that for the last two time points (24 h and 48 h) there was no clustering across each group, possibly indicating the smaller effect of duration of drug’s action as opposed to its effect.

From 41 endogenous compounds detected in the intracellular material, 16 were significantly altered and met the criteria of both univariate (*p*-value) and multivariate statistical analysis (VIP) when evaluating only cocaine treatment regardless of the time.

It was exhibited that cocaine altered the amino acid metabolism of treated cells with alanine, aspartate, and glutamate metabolic pathway being the most prominent due to the decreased intracellular levels of L-glutamate, L-alanine, L-aspartate, L-glutamine, and N-acetyl-L-aspartate. The altered levels of taurine and hypotaurine along with L-glutamine and L-alanine could be indicative of an effected taurine and hypotaurine metabolism. L-glutamate could also be attributed to arginine and proline metabolism in combination to altered L-proline and creatine and creatinine levels. L-aspartate, creatine, and betaine are involved in glycine, serine, and threonine metabolism derived by pathway analysis using KEGG [38]. Anthranillate was the only significantly differentiated compound related to tryptophan metabolism. Thymidine and uracil could probably indicate an effect of cocaine in pyrimidine metabolism. Finally reduced levels of acetyl-L-carnitine and pyridoxine were also observed upon cocaine treatment.

The present findings come in accordance with previous in vivo and in vitro studies employing metabolomics-bases analysis and enrich the panel of compounds affected by cocaine administration.

Cocaine-induced metabolome alterations in plasma of DSM-IV-diagnosed cocaine-dependent individuals were demonstrated by Patkar et al. [9,25]. Chronic exposure to cocaine resulted in higher levels of n-methylserotonin and lower levels of anthranilate attributed to perturbed tryptophan metabolism while higher levels of guanine and lower levels of hypoxanthine and xanthine to purine catabolism [25]. 

^1^H-ΝΜR metabonomics by Li et al. revealed significant regionally specific changes of cerebral g-aminobutyrate, glutamate, taurine, N-acetyl-aspartate, lactate, creatine as well as choline, phosphocholine, and glycerol in repeatedly cocaine-treated rats [26]. These endogenous metabolic variations exhibit cocaine’s effect on mitochondria dysregulation and membrane disruption [26]. Regionally specific alterations of rat neuronal metabolites were also observed by Kaplan et al. using ion mobility–mass spectrometry [39]. Nine affected metabolites involved in glycolytic pathway demonstrate the predominant effect of even a single cocaine administration [39]. 

Urine and plasma GC-MS-based metabolomics of cocaine-induced conditioned place preference (CPP) in drug-addicted rat models were conducted by Zaitsu et al. [24]. The effect of treatment was not captured in urine samples while elevation of threonine, cystine, and spermidine levels were observed in addicted rat plasma profiles [24]. 

A time-dependent effect of cocaine treatment that was attributed to the accumulation of long-chain acylcarnitines and the perturbation of several phospholipid species in murine serum and hepatic samples was shown after lipidomic profiling [27]. The induced hepatotoxicity evidenced by the impaired triacylglycerol turnover during the three days of cocaine treatment as reported by Shi et al. [27]. 

Concurrent use of intravenous self-administration of cocaine and ethanol was studied by Marcos et al. evaluating capillary electrophoresis (CE) with laser-induced fluorescence (LIF) obtained plasma amino acid profiles of a rat model [18]. Sex and treatment-related differences were detected in amino acids precursors of catecholamines such as phenylalanine and tyrosine [18]. The plasma LC-QTOF-MS profiling of rat chronically exposed to cocaine and ethanol demonstrated cocaine-triggered decreased levels of argininosuccinic acid, cystathionine, and N-ε-acetyl-L-lysine and increased levels of methionine. These results were associated to liver injury mediated by nitric oxide and ROS [40]. 

In vitro models investigating cocaine toxicity revealed that its activity at CNS was related to interaction with dopamine transporters and consequently to dopamine’s accumulation at the synaptic terminals. Generation of ROS and therefore induced oxidative stress was derived by dopamine’ auto oxidation [8]. Furthermore, the induction of cell death by apoptosis and a collapsing cellular entity compose cocaine’s cytotoxic effect on the C6 glial cells [8]. 

The involvement of an increased glutamatergic transmission in cocaine neurotoxic activity was investigated by Yablonsky-Alter et al. who concluded that the augmented release of taurine in response to glutamate-mediated excitotoxicity could act as an endogenous protective mechanism in mammal brain to chronic cocaine ingestion [41].

Cell medium samples were statically evaluated in the same manner with intracellular material. From both statistical approaches (univariate and multivariate) 30 metabolites were differentiated according to the treatment as illustrated in PCA and OPLS-DA score plots. Interestingly 9 significantly decreased metabolites in intracellular matrix were increased in the cell medium. The adverse trend could be attributed to differences in the uptake and excretion of metabolites under the effect of cocaine in HepG2 cells. Among the altered metabolites, a perpetual interchange between cellular endo-metabolome and exo-metabolome was responsible for the obtained metabolic profiles of the two types of samples. Cocaine-derived cytotoxicity potentially affected mitochondria membrane and even perturbed mitochondrial respiration [35] in the administrated dose, although only speculation about the mechanism could be made. 

A perturbed hepatic metabolism may be related to the hepatic production of toxic cocaine metabolites and subsequently to ROS during cytochrome P450-mediated oxidation. The metabolic consequences of oxidative stress include lipid peroxidation, functional, and structural changes in the biological membranes and cell death and disruption of the GSH/GSSG redox cycle [3].

Although metabolomics have proven to be a useful tool in toxicology, the elucidation of the direct mechanism through which cocaine affects cell endogenous metabolism is challenging and difficult to be answered without a multi-omics approach. The study is limited to a targeted panel of endogenous metabolites and mainly includes amino acids and related derivatives. A non-targeted metabolomics-based analysis of the samples could certainly expand the possibilities of unraveling the hidden mechanism. Metabolomics-based biomarkers well associated with oxidative stress- lipid peroxidation, could provide further information deciphering the cocaine’s cytotoxic mechanism. 

## 4. Materials and Methods

### 4.1. Reagents

Cocaine.HCl powder was purchased from Lipomed AG (Arlesheim, Switzerland). Trypsin-EDTA 1× in PBS without calcium, magnesium, and with phenol red—100 mL, penicillin-streptomycin solution 100×, fetal bovine serum (heat-inactivated)—500 mL, DMEM low glucose, w/L-glutamine, w/Na pyruvate—500 mL, 0.5% trypan blue were obtained from Biosera (Nuaille, France). XTT cell proliferation assay kit was obtained from Abnova (Taipei City, Taiwan). The HepG2 cell line was provided by Dr. George Moschialos [42].

### 4.2. Cell Culture Growth

HepG2 cells were cultivated in flasks with 10 mL of DMEM containing 10% FBS, 1% glutamine, and 1% PS at 37 °C under 5% CO_2_ and humidified atmosphere. Three days after, cell medium was replaced by a freshly prepared medium. When the flask was coherent cell medium was aspirated and cells were washed twice with 6–7 mL PBS solution. Then 3 mL trypsin was added and left for 1 to 2 min for the cells to be detached from the flask. Trypsin was deactivated with the addition of 7 mL of the medium. About 100 μL of the medium mixture was then mixed with 100 μL of trypan blue solution and 20 μL was transferred to a hemocytometer. After cell count, the mixture was centrifuged for 5 min at 1000 rpm and the supernatant was collected. About 3 mL of fresh medium was then added, and the preferred population of cells was transferred to a new flask or to 6-well plates for further incubation for growth/cultivation. 

### 4.3. XTT Assay

For the evaluation of lethal concentration, IC_50_ was experimentally calculated with an XTT Cell Proliferation Assay Kit. The cytotoxicity study was conducted on cocaine exposed HepG2 cells in 96-well plates. Specifically, 100 μL of medium containing 10^4^ cells were seeded in each well. The cells were left for one day to acclimate prior to cocaine exposure. Then they were treated with different cocaine concentrations by the addition of 1 μL of cocaine solution in PBS. The final concentrations of cocaine in the wells were 0.5 mM, 1 mM, 2 mM, 5 mM, and 10 mM. The cells were incubated for 24 h and afterwards 10 μL of the prepared XTT mixture was added to each well followed by gentle mixing with an orbital shaker and left for two hours. After they were mixed gently for one minute to ensure homogenous distribution of color, the absorbance of each sample was measured using an Awareness Technologies Stat Fax 2100 Microplate Reader (Palm City, FL, USA) at a wavelength of 450 nm and also at 630 nm for matrix correction.

### 4.4. Cell Growth Rate

To study the cell growth rate, (2.5 × 10^4^) 1.3 × 10^4^ cells were seeded in each well at a 24-well plate in 0.5 mL of medium and left for one day to acclimate at 37 °C under 5% CO_2_ and humidified atmosphere. Twelve wells were treated with cocaine (addition of 5 μL of 200 mM cocaine in PBS 1% to final 2 mM final concentration) while in twelve others same volume of neat PBS was added and used as control samples. For each time point, three control and three treated samples were used. The cell growth was evaluated at 3 h, 24 h, 48 h, and 72 h after cocaine administration. To measure the cell growth rate, cell medium was removed, wells were washed with 1% PBS solution followed by the addition of 0.1 mL of 0.25% trypsin-EDTA solution for 1 min. As a next step, 0.4 mL of cell medium was added to deactivate any metabolic activity, the mix was carefully vortexed and 0.05 mL mixed with 0.05 mL of 0.4% trypan blue dye, loaded on hemocytometer, and finally examined under a microscope at low magnification.

### 4.5. Metabolic Profiling Analysis 

The cell line was treated as described in paragraph Cell culture growth. For the metabolomic analysis 3 × 10^5^ cells per well were seeded in a 6-well plate. Three control and three treated samples with cocaine were prepared for each time point. The experiment consisted of three different time points, so a total of 9 control vs. 9 cocaine-treated wells (samples) were prepared. Specifically, the control samples were treated with 20 μL of PBS 1% and the treated samples were spiked with 20 μL of 200 mM cocaine in PBS 1% (for a final concentration of 2 mM). Then the samples were left at 37 °C under 5% CO_2_ and humidified atmosphere.

### 4.6. Sample Preparation

The whole cell medium was collected and centrifuged at 10,000 rpm for 10 min and the supernatant was stored at −24 °C. Following medium aspiration, cells were washed twice with 1 mL of PBS solution to remove the remaining medium. Afterwards 2 mL of ACN: MeOH: H_2_O 50:30:20 (*v*/*v*) was added to the wells, and they were placed in the freezer for 20 min. After cells were scraped and vortexed, the extract was collected in an Eppendorf tube. The mixture was then centrifuged at 10,000 rpm and −4 °C for 10 min and the supernatant was collected and dried using a speedvac evaporator. Reconstitution was performed with 300 μL MeOH. 

For the metabolomics analysis of the intracellular material, 15 μL of the reconstituted extract was mixed with 15 μL H_2_O and 70 μL ACN and placed in the autosampler. 

For the metabolomics analysis of the cell culture medium, 15 μL of the sample were mixed with 15 μL MeOH and 70 μL can. Samples were then centrifuged (10 min, 4 °C, 11,800× *g*) and the supernatant was transferred to a LC-MS vial for analysis. 

### 4.7. LC-MS/MS Analysis 

Metabolic profiling was performed with a targeted LC-MS/MS validated method [28] which provide quantitative data for 106 low molecular weight metabolites. An Acquity UPLC system with a triple quadruple (Xevo TQD system, Waters Corp, Millford, MA, USA) was implemented. An Acquity BEH Amide Column (2.1 mm × 150 mm, 1.7 μm), (Waters Ltd., Elstree, UK) at 40 °C and with a mobile phase of A: 95:5 *v*/*v* ACN/H_2_O and B: 70:30 *v/v* H2O/ACN, both contained 10 mM ammonium formate under gradient elution was used. MS was operating at positive and negative ionization mode using electron spray ionization (ESI). 

The samples were analyzed in a randomized order in one analytical batch. Quantification was based on a mean of two calibration curves series analyzed at the beginning and at the end of the analytical batch. A blank sample was prepared by mixing 15 μL H_2_O, 15 μL MeOH, and 70 μL ACN and was analyzed before and after the calibration curves. Details on the metabolites concentration ranges of the analytical standard solutions can be found in previously published work from our group [28]. To assess system’s analytical stability, QC samples were prepared by mixing a 20 μL aliquot of all samples (9 treated and 9 control samples). The QC samples for both cell medium and intracellular material were prepared the same way as the real samples and were analyzed as follows: two QC samples were analyzed in the beginning of the analytical batch (equilibration of the system to the matrix), then every after 6 samples and at the end of the batch. A total of six QCs were analyzed for each matrix.

### 4.8. Data Handling

For the data acquisition and treatment Waters MassLynx v. 4.1. and TargetLynx were used. Multivariate statistics and other chemometric analysis were performed by SimcaP 13 (Umetrics, Umea, Sweden). Metaboanalyst was used for the calculation of AUC-ROC and log2FC values. 

## Figures and Tables

**Table 1:** Metabolites which were detected in the intracellular material and their *p*-value and fold change value at each time point; the compounds are listed based on their *p*-value at 48 h (cocaine vs. control), increasing from top to bottom.

		Control (μg/mL)			Cocaine (μg/mL)			*p*-Value			Log2FC (Cocaine vs. Control)		
No.	Compound	3 h	24 h	48 h	3 h	24 h	48 h	3 h	24 h	48 h	3 h	24 h	48 h
1	Betaine	0.129	0.368	0.768	0.090	0.096	0.175	3 × 10^−2^	4 10^−2^	**7 × 10^−5^**	−0.51	−1.93	−2.14
2	Hypotaurine	6.619	11.14	12.59	2.592	5.821	3.796	8 × 10^−2^	10^−1^	**6 × 10^−3^**	−1.35	−0.94	−1.73
3	Acetylcarnitine	0.041	0.068	0.107	0.032	0.004	0.006	9 × 10^−2^	7 × 10^−3^	**6 × 10^−3^**	−0.33	−4.08	−4.12
4	Creatine	0.560	1.107	1.994	0.419	0.314	0.763	9 × 10^−3^	10^−2^	**7 × 10^−3^**	−0.42	−1.82	−1.39
5	Acetyl aspartate	0.826	2.946	3.929	0.678	0.794	0.940	7 × 10^−1^	2 × 10^−1^	**10^−2^**	−0.28	−1.89	−2.06
6	Serine	1.886	2.469	4.012	1.449	3.025	0.584	6 × 10^−1^	8 × 10^−1^	**10^−2^**	−0.38	0.29	−2.78
7	Glutamine	11.02	16.29	20.88	11.44	8.056	8.182	8 × 10^−1^	2 × 10^−2^	**2 ×10^−2^**	0.05	−1.02	−1.35
8	Taurine	1.064	1.717	2.619	0.699	1.103	1.220	5 × 10^−2^	5 × 10^−2^	**3 × 10^−2^**	−0.61	−0.64	−1.10
9	Alanine	1.534	2.447	3.840	1.599	1.846	1.649	8 × 10^−1^	2 × 10^−1^	**3 × 10^−2^**	0.06	−0.41	−1.22
10	Proline	1.010	1.661	1.972	0.632	0.712	0.818	3 × 10^−1^	3 × 10^−2^	**4 × 10^−2^**	−0.68	−1.22	−1.27
11	Anthranilic acid	0.440	1.527	1.959	0.212	0.552	0.314	5 × 10^−1^	2 × 10^−1^	**4 × 10^−2^**	−1.06	−1.47	−2.64
12	Creatinine	0.060	0.099	0.116	0.054	0.064	0.064	2 × 10^−1^	3 × 10^−2^	**4 × 10^−2^**	−0.16	−0.63	−0.86
13	Pyridoxine	0.028	0.027	0.027	0.025	0.014	0.016	4 × 10^−2^	3 × 10^−3^	**4 × 10^−2^**	−0.21	−0.93	−0.80
14	Glutamic acid	8.255	15.979	29.92	7.682	8.928	11.21	7 × 10^−1^	10^−1^	**4 × 10^−2^**	−0.10	−0.84	−1.42
15	Pantothenate	0.148	0.196	0.378	0.210	0.135	0.152	5 × 10^−2^	8 × 10^−2^	5 × 10^−2^	0.50	−0.54	−1.31
16	Aspartic acid	3.073	4.953	6.476	2.935	3.503	2.213	7 × 10^−1^	5 × 10^−1^	6 × 10^−2^	−0.07	−0.50	−1.55
17	Uracil	0.029	0.043	0.046	0.015	0.027	0.021	8 × 10^−2^	8 × 10^−2^	8 × 10^−2^	−0.93	−0.63	−1.11
18	Choline	0.003	0.009	0.007	0.006	0.005	0.004	4 × 10^−3^	10^−1^	8 × 10^−2^	1.25	−0.79	−0.92
19	Isoleucine	0.315	0.858	1.148	0.384	0.416	0.612	8 × 10^−1^	9 × 10^−2^	8 × 10^−2^	0.28	−1.04	−0.91
20	Benzoic acid	0.417	0.462	0.428	0.386	0.339	0.604	8 × 10^−1^	3 × 10^−1^	9 × 10^−2^	−0.11	−0.45	0.50
21	leucine	0.577	1.283	1.466	0.665	0.861	0.887	6 × 10^−1^	3 × 10^−1^	10^−1^	0.20	−0.58	−0.73
22	Threonine	1.818	2.626	3.498	1.901	2.246	2.115	8 × 10^−1^	7 × 10^−1^	10^−1^	0.06	−0.23	−0.73
23	Cytosine	0.047	0.047	0.046	0.046	0.046	0.046	5 × 10^−1^	4 × 10^−1^	10^−1^	−0.03	−0.03	−0.01
24	Adenine	0.018	0.022	0.025	0.021	0.021	0.021	5 × 10^−3^	7 × 10^−1^	10^−1^	0.18	−0.06	−0.26
25	Nicotinamide	0.008	0.061	0.084	0.010	0.041	0.036	5 × 10^−1^	4 × 10^−1^	10^−1^	0.36	−0.55	−1.24
26	Asparagine	0.938	0.961	0.935	0.900	0.926	0.854	6 × 10^−1^	7 × 10^−1^	10^−1^	−0.06	−0.05	−0.13
27	Thymidine	0.007	0.009	0.007	0.004	0.003	0.004	5 × 10^−2^	4 × 10^−2^	10^−1^	−0.91	−1.53	−0.76
28	Tyrosine	0.958	2.024	2.663	1.326	1.542	2.146	3 × 10^−1^	2 × 10^−1^	2 × 10^−1^	0.47	−0.39	−0.31
29	Pyroglutamic	2.749	3.503	5.955	2.114	4.744	3.479	7 × 10^−1^	4 × 10^−1^	2 × 10^−1^	−0.38	0.44	−0.78
30	Norvaline-Valine	1.110	1.625	1.936	1.155	1.401	1.407	9 × 10^−1^	4 × 10^−1^	3 × 10^−1^	0.06	−0.21	−0.46
31	Glycine	1.307	2.570	4.963	1.350	2.064	3.606	9 × 10^−1^	3 × 10^−1^	3 × 10^−1^	0.05	−0.32	−0.46
32	Methionine	0.148	0.338	0.400	0.155	0.159	0.250	9 × 10^−1^	9 × 10^−2^	3 × 10^−1^	0.07	−1.09	−0.68
33	Phenylalanine	1.058	1.506	1.455	1.094	1.325	1.276	8 × 10^−1^	3 × 10^−1^	4 × 10^−1^	0.05	−0.19	−0.19
34	Sorbitol	1.629	2.422	3.273	2.752	1.676	1.980	4 × 10^−1^	4 × 10^−1^	4 × 10^−1^	0.76	−0.53	−0.73
35	Lactic acid	13.46	15.17	14.10	8.273	14.62	17.44	10^−1^	9 × 10^−1^	5 × 10^−1^	−0.70	−0.05	0.31
36	Lysine	1.270	1.713	1.981	1.214	1.430	1.667	4 × 10^−1^	3 × 10^−1^	5 × 10^−1^	−0.07	−0.26	−0.25
37	Thiamine	0.085	0.091	0.091	0.083	0.087	0.088	10^−1^	2 × 10^−1^	5 × 10^−1^	−0.03	−0.06	−0.05
38	Tryptophan	0.803	0.814	0.938	0.806	0.831	0.850	9 × 10^−1^	6 × 10^−1^	5 × 10^−1^	0.01	0.03	−0.14
39	Ornithine	1.194	1.184	1.270	1.178	1.116	1.169	8 × 10^−1^	9 × 10^−1^	7 × 10^−1^	−0.02	−0.09	−0.12
40	Mannitol	1.775	1.444	1.492	1.172	1.353	1.606	10^−1^	7 × 10^−1^	7 × 10^−1^	−0.60	−0.09	0.11
41	Arginine	0.210	0.202	0.221	0.140	0.620	0.207	3 × 10^−1^	4 × 10^−1^	8 × 10^−1^	−0.58	1.62	−0.09

**Table 2:** Metabolites that were detected in cell medium and their *p*-value and fold change value at each time point; the compounds are listed based on their *p*-values at 48 h (cocaine vs. control), increasing from top to bottom.

		Control (μg/mL)			Cocaine (μg/mL)			*p*-Value			Log2FC (Cocaine vs. Control)		
No.	Compound	3 h	24 h	48 h	3 h	24 h	48 h	3 h	24 h	48 h	3 h	24 h	48 h
1	Benzoic acid	0.190	0.215	0.254	7.662	14.26	18.63	10^−3^	2 × 10^−7^	**3 × 10^−4^**	5.33	6.05	6.19
2	Choline	0.124	0.072	0.032	0.189	0.198	0.165	3 × 10^−3^	10^−8^	**4 × 10^−4^**	0.61	1.46	2.37
3	Aspartic acid	2.039	1.965	2.770	2.379	5.397	6.420	4 × 10^−1^	5 × 10^−4^	**2 × 10^−3^**	0.22	1.46	1.21
4	Malonic acid	5.296	5.028	4.955	5.234	3.444	5.380	8 × 10^−1^	6 × 10^−1^	**4 × 10^−3^**	−0.02	−0.55	0.12
5	Thymidine	0.041	0.022	0.015	0.066	0.051	0.029	2 × 10^−2^	10^−3^	**8 × 10^−3^**	0.69	1.24	0.93
6	Uridine	0.061	0.060	0.086	0.141	0.236	0.326	10^−5^	5 × 10^−3^	**2 × 10^−2^**	1.21	1.97	1.93
7	Phenylalanine	26.10	19.32	25.03	38.31	49.78	49.09	10^−1^	4 × 10^−5^	**2 × 10^−2^**	0.55	1.37	0.97
8	Hypoxanthine	0.115	0.032	0.007	0.200	0.272	0.317	2 × 10^−3^	8 × 10^−5^	**3 × 10^−2^**	0.80	3.07	5.52
9	Hippuric acid	0.356	0.409	0.518	0.432	0.672	0.608	5 × 10^−1^	10^−1^	**3 × 10^−2^**	0.28	0.72	0.23
10	Mannitol	3.006	2.391	4.760	5.075	5.086	6.759	5 × 10^−2^	4 × 10^−2^	**4 × 10^−2^**	0.76	1.09	0.51
11	Creatine	1.606	1.766	2.209	2.472	3.364	3.447	3 × 10^−2^	5 × 10^−4^	**4 × 10^−2^**	0.62	0.93	0.64
12	Glutamic acid	29.35	35.92	60.79	37.60	85.88	134.8	3 × 10^−1^	3 × 10^−4^	5 × 10^−2^	0.36	1.26	1.15
13	Glycine	22.47	28.00	38.30	34.20	48.31	60.63	4 × 10^−2^	8 × 10^−3^	5 × 10^−2^	0.61	0.79	0.66
14	Alanine	10.21	19.83	35.71	13.47	38.74	48.84	10^−1^	10^−3^	8 × 10^−2^	0.40	0.97	0.45
15	Asparagine	1.623	2.150	3.106	1.985	3.849	5.670	6 × 10^−2^	10^−3^	8 × 10^−2^	0.29	0.84	0.87
16	Arginine	23.84	23.28	30.03	44.15	51.26	47.10	3 × 10^−2^	5 × 10^−6^	8 × 10^−2^	0.89	1.14	0.65
17	Xanthine	0.342	0.371	0.631	0.592	0.893	1.167	5 × 10^−3^	3 × 10^−3^	9 × 10^−2^	0.79	1.27	0.89
18	Acetylcarnitine	0.049	0.048	0.065	0.067	0.088	0.092	9 × 10^−2^	3 × 10^−4^	10^−1^	0.45	0.86	0.51
19	Homocysteine	14.37	21.51	31.46	14.29	42.48	50.90	10^0^	8 × 10^−2^	10^−1^	−0.01	0.98	0.69
20	Norvaline-Valine	40.54	35.39	47.70	55.02	66.90	67.49	10^−1^	8 × 10^−5^	10^−1^	0.44	0.92	0.50
21	Methionine	13.19	10.56	14.68	18.00	21.98	21.27	7 × 10^−2^	3 × 10^−4^	10^−1^	0.45	1.06	0.53
22	Tryptophan	6.289	4.624	7.233	8.632	10.94	11.06	2 × 10^−1^	8 × 10^−5^	10^−1^	0.46	1.24	0.61
23	Taurine	1.001	1.032	1.016	1.453	1.484	1.309	10^−1^	10^−1^	10^−1^	0.54	0.52	0.37
24	Lysine	59.84	53.75	76.39	89.80	110.5	111.4	6 × 10^−2^	6 × 10^−5^	10^−1^	0.59	1.04	0.54
25	Uracil	0.331	0.374	0.570	0.636	0.724	0.837	4 × 10^−2^	6 × 10^−4^	10^−1^	0.95	0.95	0.55
26	Isoleucine	57.14	46.12	62.89	84.93	102.6	94.04	7 × 10^−2^	2 × 10^−5^	10^−1^	0.57	1.15	0.58
27	Thymine	0.030	0.062	0.095	0.052	0.092	0.133	2 × 10^−1^	4 × 10^−3^	10^−1^	0.79	0.57	0.49
28	Adenine	0.018	0.017	0.017	0.018	0.019	0.018	6 × 10^−1^	7 × 10^−3^	10^−1^	0.07	0.16	0.11
29	leucine	40.98	33.85	46.79	64.32	77.14	68.77	7 × 10^−2^	5 × 10^−5^	2 × 10^−1^	0.65	1.19	0.56
30	Lactic acid	476.4	612.1	879.3	552.9	1067	1230	4 × 10^−1^	3 × 10^−3^	2 × 10^−1^	0.21	0.80	0.48
31	Pantothenate	1.650	1.376	2.288	2.444	3.062	3.134	7 × 10^−2^	2 × 10^−4^	2 × 10^−1^	0.57	1.15	0.45
32	Anthranilic acid	6.135	8.028	20.20	8.463	18.48	31.89	4 × 10^−2^	5 × 10^−3^	2 × 10^−1^	0.46	1.20	0.66
33	Tyrosine	32.09	31.30	52.33	55.24	61.81	78.54	2 × 10^−2^	2 × 10^−3^	2 × 10^−1^	0.78	0.98	0.59
34	Creatinine	1.413	1.313	1.961	1.994	2.483	2.569	6 × 10^−2^	6 × 10^−5^	2 × 10^−1^	0.50	0.92	0.39
35	Allontoin	1.506	1.426	2.182	2.306	2.677	2.802	5 × 10^−2^	3 × 10^−4^	2 × 10^−1^	0.62	0.91	0.36
36	Threonine	29.51	28.00	40.98	42.43	51.64	52.87	10^−1^	2 × 10^−4^	2 × 10^−1^	0.52	0.88	0.37
37	Betaine	1.229	1.157	1.640	1.641	1.988	2.069	6 × 10^−2^	8 × 10^−4^	2 × 10^−1^	0.42	0.78	0.33
38	Folic acid	2.834	3.069	3.110	3.206	4.177	4.247	6 × 10^−1^	3 × 10^−2^	2 × 10^−1^	0.18	0.44	0.45
39	Sorbitol	5.067	6.675	9.881	5.629	6.356	13.84	9 × 10^−1^	9 × 10^−1^	3 × 10^−1^	0.15	−0.07	0.49
40	Pyroglutamic	92.66	105.46	161.7	139.1	175.1	201.1	6 × 10^−2^	6 × 10^−4^	3 × 10^−1^	0.59	0.73	0.31
41	Ornithine	4.183	6.162	11.27	7.507	10.65	14.07	4 × 10^−3^	4 × 10^−3^	3 × 10^−1^	0.84	0.79	0.32
42	Pyruvic acid	4.507	4.636	6.377	5.944	7.868	8.150	10^−1^	10^−3^	3 × 10^−1^	0.40	0.76	0.35
43	Nicotinamide	2.225	2.097	2.910	2.624	3.322	3.548	10^−1^	5 × 10^−4^	3 × 10^−1^	0.24	0.66	0.29
44	Fructose	3.865	1.175	n.d. *	6.063	3.015	0.100	4 × 10^−2^	3 × 10^−3^	4 × 10^−1^	0.65	1.36	−
45	Serine	17.06	14.44	18.50	20.58	20.60	16.16	10^−1^	4 × 10^−2^	5 × 10^−1^	0.27	0.51	−0.20
46	Glutamine	236.3	187.8	222.6	332.1	322.4	252.6	6 × 10^−2^	10^−4^	5 × 10^−1^	0.49	0.78	0.18
47	Pyridoxine	0.007	0.013	0.041	0.009	0.027	0.037	3 × 10^−1^	2 × 10^−3^	7 × 10^−1^	0.47	0.98	−0.14
48	Acetyl aspartate	0.166	0.193	0.406	0.161	0.293	0.340	8 × 10^−1^	3 × 10^−1^	8 × 10^−1^	−0.04	0.60	−0.26
49	Inositol	13.43	12.06	13.79	12.10	12.86	14.71	8 × 10^−1^	9 × 10^−1^	8 × 10^−1^	−0.15	0.09	0.09
50	Trimethylamine-n-oxide	0.050	0.041	0.056	0.058	0.065	0.058	5 × 10^−1^	9 × 10^−4^	8 × 10^−1^	0.20	0.65	0.05
51	Cytosine	0.049	0.048	0.050	0.049	0.049	0.051	9 × 10^−1^	4 × 10^−1^	8 × 10^−1^	0.01	0.05	0.02
52	Proline	2.390	4.543	10.499	2.998	6.574	10.14	2 × 10^−1^	5 × 10^−3^	8 × 10^−1^	0.33	0.53	−0.05
53	Thiamine	0.103	0.206	0.241	0.104	0.208	0.247	8 × 10^−1^	8 × 10^−1^	9 × 10^−1^	0.01	0.02	0.03
54	Glucose	261.21	101.07	8.194	352.0	154.1	7.714	2 × 10^−2^	3 × 10^−2^	10^0^	0.43	0.61	−0.09
55	Mannose	48.812	20.502	n.d. *	75.442	35.656	n.d. *	9 × 10^−3^	5 × 10^−3^	-	0.63	0.80	−

* n.d.: not detected.

**Table 3:** Model characteristics of PCA and OPLS-DA multivariate analysis.

Comparisons	Analysis	R^2^X	R^2^Y	Q^2^Y
Control vs. cocaine-treated vs. QC (intracellular material)	PCA	0.604		0.388
Control vs. cocaine-treated (intracellular material)	PCA	0.644		0.417
Control vs. cocaine-treated (intracellular material)	OPLS-DA	0.638	0.859	0.685
Control vs. cocaine-treated vs. QC (cell medium)	PCA	0.800		0.728
Control vs. cocaine-treated (cell medium)	PCA	0.806		0.726
Control vs. cocaine-treated (cell medium)	OPLS-DA	0.800	0.835	0.765

## Supplementary Materials

The following are available online. Figure S1. The structure of cocaine. Figure S2. PCA model showing QC samples in blue and control and cocaine-treated intracellular material samples in grey. Figure S3. OPLS-DA model showing control (blue) and cocaine-treated (red) intracellular samples which are discriminated clearly (all control samples in the left side and all the treated in the right side). Figure S4. PCA model showing QC (blue) and control, cocaine-treated cell medium samples (grey). Figure S5. OPLS-DA model showing control (blue) and cocaine-treated (red) cell medium samples.

## Sample Availability

Samples of the compounds are not available from the authors.
