# Supplementary material for: Evaluation of Cocaine Effect on Endogenous Metabolites of HepG2 Cells Using Targeted Metabolomics
Source: Molecules. 2021 Jul 29;26(15):4610. doi: 10.3390/molecules26154610 (PMC8347943; doi:10.3390/molecules26154610)
Supplement: Supplementary file 1 [file molecules-26-04610-s001.zip › molecules-1308442-supplementary.pdf]

Evaluation of Cocaine Effect on Intracellular Small Polar Metabolites of Hepg2 Cells Using Targeted Metabolomics

Adamantios Krokos [1], Olga Deda [2,3], Christina Virgiliou [1,3], Helen Gika [2,3], Nikolaos Raikos [2], Eleni Aggelidou [4], Aristidis Kritis [4], Georgios Theodoridis [1,3*]

1 Department of Chemistry, Aristotle University of Thessaloniki, 54124 Thessaloniki, Greece; akrokosa@chem.auth.gr (A.K.), cr_virgi@hotmail.com (C.V.)
2 Laboratory of Forensic Medicine and Toxicology, Department of Medicine, Aristotle University of Thessaloniki, 54124 Thessaloniki, Greece; oliadmy@gmail.com (O.D.), gkikae@auth.gr (H.G.), raikos@auth.gr (N.R.)
3 Biomic_Auth, Bioanalysis and Omics Lab, B 1.4 Centre for Interdisciplinary Research of Aristotle University of Thessaloniki, Innovation Area of Thessaloniki, Balkan Center, Greece
4 Laboratory of Physiology and Pharmacology, School of Medicine, Aristotle University of Thessaloniki, Thessaloniki, 54124 Greece; angelide@auth.gr (E.A.), kritis@auth.gr (A.K.)
* Correspondence: gtheodor@chem.auth.gr ; Tel.: +30 2310 997718

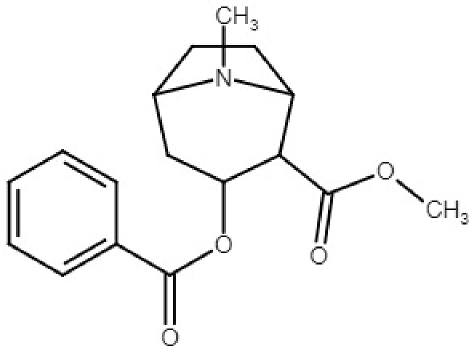

Figure S1. The structure of cocaine.

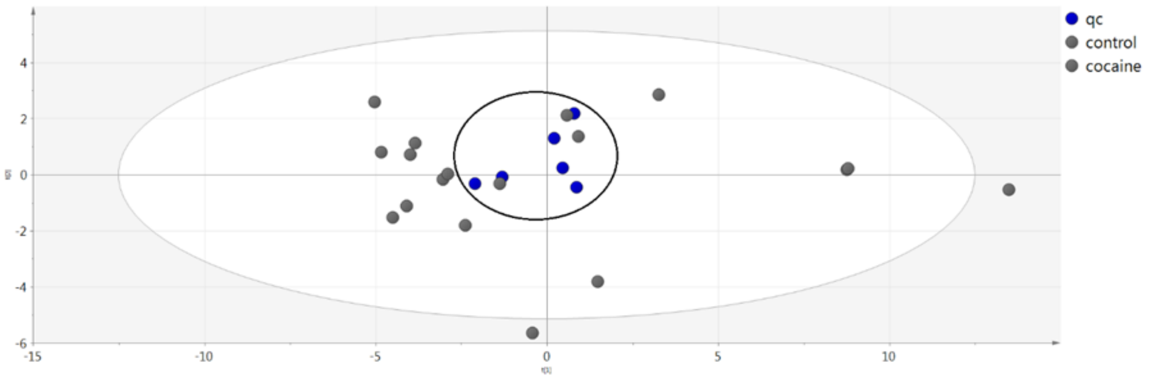

Figure S2. PCA model showing QC samples in blue and control and cocaine treated intracellular material samples in gray.

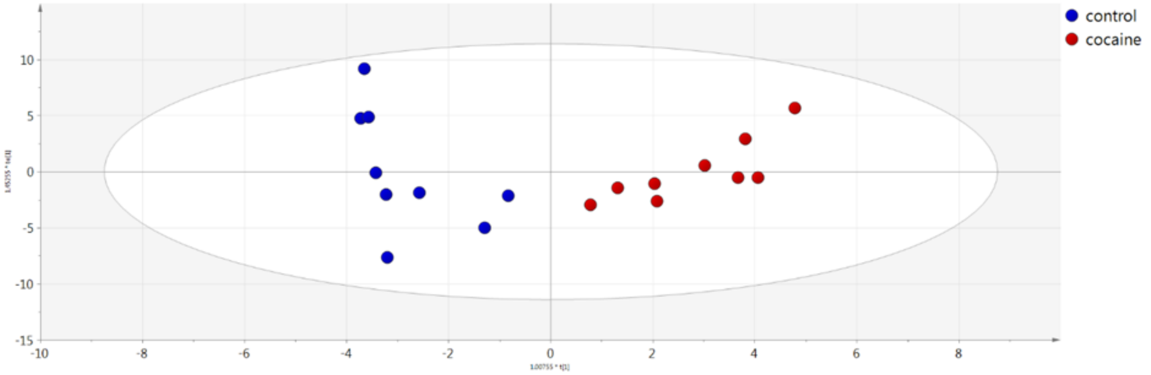

Figure S3. OPLS-DA model showing control (blue) and cocaine treated (red) intracellular samples which are discriminated clearly (all control samples in the left side and all the treated in the right side).

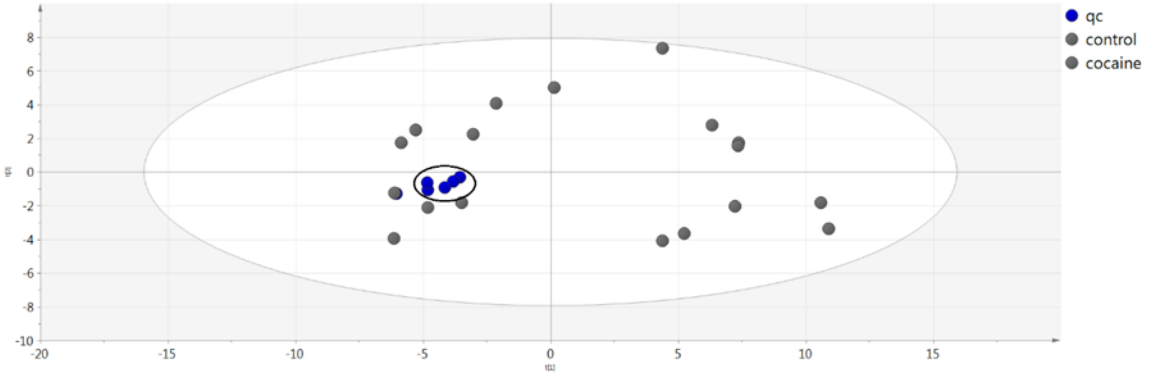

Figure S4. PCA model showing QC (blue) and control, cocaine treated cell medium samples (gray).

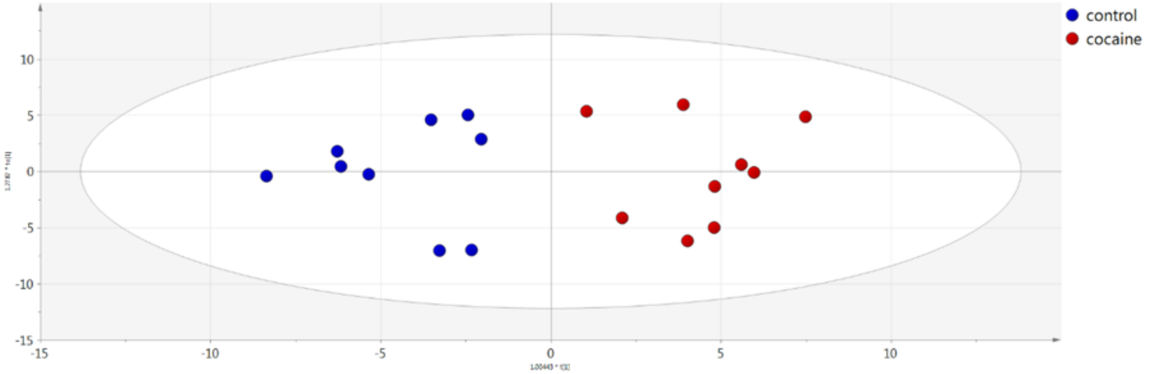

Figure S5. OPLS-DA model showing control (blue) and cocaine treated (red) cell medium samples.
